# Amplified Fragments of an Autosome-Borne Gene Constitute a Significant Component of the W Sex Chromosome of *Eremias velox* (Reptilia, Lacertidae)

**DOI:** 10.3390/genes12050779

**Published:** 2021-05-20

**Authors:** Artem Lisachov, Daria Andreyushkova, Guzel Davletshina, Dmitry Prokopov, Svetlana Romanenko, Svetlana Galkina, Alsu Saifitdinova, Evgeniy Simonov, Pavel Borodin, Vladimir Trifonov

**Affiliations:** 1 Institute of Environmental and Agricultural Biology (X-BIO), University of Tyumen, Lenina str. 23, 625003 Tyumen, Russia; e.p.simonov@utmn.ru; 2 Institute of Cytology and Genetics SB RAS, Acad. Lavrentiev Ave. 10, 630090 Novosibirsk, Russia; guzel@mcb.nsc.ru (G.D.); borodin@bionet.nsc.ru (P.B.); 3 Institute of Molecular and Cellular Biology SB RAS, Acad. Lavrentiev Ave. 8/2, 630090 Novosibirsk, Russia; ada@mcb.nsc.ru (D.A.); dprokopov@mcb.nsc.ru (D.P.); rosa@mcb.nsc.ru (S.R.); vlad@mcb.nsc.ru (V.T.); 4 Department of Genetics and Biotechnology, Saint Petersburg State University, Universitetskaya Emb. 7–9, 199034 Saint Petersburg, Russia; svetlana.galkina@spbu.ru; 5 Department of Human and Animal Anatomy and Physiology, Herzen State Pedagogical University of Russia, Moyka Emb. 48, 191186 Saint Petersburg, Russia; saifitdinova@mail.ru; 6 Novosibirsk State University, Pirogova str. 3, 630090 Novosibirsk, Russia

**Keywords:** sex chromosomes, heterochromatin, ATF7IP2, repetitive DNA, lizards

## Abstract

Heteromorphic W and Y sex chromosomes often experience gene loss and heterochromatinization, which is frequently viewed as their “degeneration”. However, the evolutionary trajectories of the heterochromosomes are in fact more complex since they may not only lose but also acquire new sequences. Previously, we found that the heterochromatic W chromosome of a lizard *Eremias velox* (Lacertidae) is decondensed and thus transcriptionally active during the lampbrush stage. To determine possible sources of this transcription, we sequenced DNA from a microdissected W chromosome sample and a total female DNA sample and analyzed the results of reference-based and de novo assembly. We found a new repetitive sequence, consisting of fragments of an autosomal protein-coding gene ATF7IP2, several SINE elements, and sequences of unknown origin. This repetitive element is distributed across the whole length of the W chromosome, except the centromeric region. Since it retained only 3 out of 10 original ATF7IP2 exons, it remains unclear whether it is able to produce a protein product. Subsequent studies are required to test the presence of this element in other species of Lacertidae and possible functionality. Our results provide further evidence for the view of W and Y chromosomes as not just “degraded” copies of Z and X chromosomes but independent genomic segments in which novel genetic elements may arise.

## 1. Introduction

Sex chromosomes originate from autosomal pairs when one chromosome acquires a dominant sex-determining locus. In some cases, the product of this locus interacts directly with the molecular physiological pathways of gonadal development, such as in mammals and in the African clawed frog (*Xenopus laevis*) [1,2]. In other cases, this locus represents a null allele and acts via haploinsufficiency mechanisms in heterozygotes, such as in birds [3]. The emergence of sex chromosomes is frequently followed by a suppression of recombination between the homologs in the heterogametic sex. The non-recombining segments gradually spread from a narrow zone around the sex determination locus to almost the whole length of the chromosomes, except the small pseudoautosomal region required for proper segregation of the sex chromosomes during meiosis [4]. This pathway was followed by a majority of sufficiently old vertebrate sex chromosome systems, although there are some systems that do not show significant spreading of the non-recombining regions over millions of years [5].

There are different explanations for this suppression of recombination. According to the most popular concept, nascent sex chromosomes tend to accumulate sexually antagonistic genes (i.e., useful for one sex but deleterious for the other), and inhibition of recombination is favored because it prevents disruption of the linkage between the sex-determining locus and the sexually antagonistic alleles [6,7]. Indeed, it is known that the Y chromosomes of mammals carry genes that are involved in testis development (for example, ZFY) [8], and sex chromosomes of guppies (*Poecilia reticulata*) carry genes responsible for male coloration [9,10]. In the subgenus *Micropoecilia*, which is closely related to guppies, male coloration is almost completely determined by the Y chromosome haplotype, and, thus, there is a limited number of distinct male color morphs linked to specific Y haplotypes [11].

The suppression of recombination between the sex chromosomes leads to a divergence between the X/Z and Y/W chromosome sequences over evolutionary time. In particular, the non-recombining Y and W chromosomes show loss of protein-coding genes, heterochromatinization, and repetitive sequence accumulation, which is generally understood as their “degradation” [12,13]. However, this term oversimplifies the evolutionary trajectories of Y and W chromosomes, which in many taxa show complex genetic content. In addition to the sexually antagonistic genes, they may contain preserved or neofunctionalized ancestral genes, homologous to the genes on X and Z chromosomes (gametologs), rDNA gene clusters, and other functional sequences [14,15,16,17,18]. Studies of genetic contents of Y and W chromosomes are important to understand their underestimated functional role in sex-specific developmental and physiological processes.

Currently, the best studied Y and W chromosomes are those of mammals and birds, and, to a lesser extent, snakes [15,17,19,20,21,22,23,24]. There are also some important studies of sex chromosomes of fish and amphibians: for example, sex-determining genes are known for medaka (XY) [25] and the African clawed frog (ZW) [2]. Sex chromosome structures and patterns of evolutionary transitions are also extensively studied in other species of frogs [26,27,28]. An extensive body of literature exists on the sex chromosomes of guppy fish [29,30,31,32]. However, for many other vertebrate Y and W chromosomes, especially in reptiles, it is only known that they do not have certain genes that their X and Z counterparts possess [33,34], and that they accumulate certain simple repeat sequences [35].

Among the vertebrate groups with insufficiently studied “degenerated” sex chromosomes, the true, or lacertid, lizards (Lacertidae) are particularly interesting. First, their sex chromosomes are partially homologous to the sex chromosomes of therian mammals [36], although the lacertids are female heterogametic (ZZ/ZW) [37]. The sex chromosomes of lacertids are homologous across the whole family, but in outgroup taxa, such as teiids and amphisbaenians, the genomic segments homologous to the lacertid ZZ/ZW chromosomes remain autosomal [38].

Second, their W chromosomes are extremely diverse in size and genetic content, in contrast with Z chromosomes. W chromosomes of various lacertid species contain telomeric repeats, microsatellites, and IMO-TaqI satellites, which are usually located in pericentromeric heterochromatic blocks on lacertid autosomes [39,40,41]. 

Recently, we studied sex chromosome behavior in meiotic prophase at the lampbrush stage in a lacertid *Eremias velox* (rapid racerunner), and obtained a microdissected W-chromosome painting probe to identify the sex bivalent at the lampbrush chromosome spread [42]. We found that the *E. velox* W chromosome at the lampbrush stage is more decondensed than autosomes and possesses numerous lateral DNA loops. The lateral loops of lampbrush chromosomes are proven to be sites of transcriptional activity [43,44,45]. This is in sharp contrast to the bird W chromosome, which is highly condensed and silenced at the lampbrush stage [46]. Considering the fact that the bulk of lampbrush lateral loops is represented by sequences that do not encode proteins [45], the W chromosome of *E.velox* should be saturated with these elements (including tandem and interspersed repeats). 

The genetic content of the W chromosome of *E. velox* was investigated previously using FISH with probes to diverse microsatellite sequences, and the accumulation of several satellite types was detected [47]. However, this approach is not suitable for uncovering unique features of W chromosomes, since it is limited to checking the presence or absence of a priori known sequences.

To investigate the genetic content of the *E. velox* W chromosome in more detail and identify possible sources of the transcription, in this study we sequenced the previously obtained W-specific probe, and analyzed it both by aligning it to the reference genome of a related species, the European wall lizard (*Podarcis muralis*), and by de novo assembly. Since microdissected chromosome probes usually contain only partial chromosomes (due to material loss during microdissection and to amplification artifacts), we also sequenced the total genomic DNA of a female *E. velox*. Since the W chromosome in this species is relatively large [42], W-derived reads should constitute a significant part of the total genomic sequences.

## 2. Materials and Methods

The samples, the methods of the preparation of mitotic and lampbrush chromosome samples, the extraction of genomic DNA, and the production of the W-specific microdissected probe were described in detail previously [42]. Briefly, mitotic chromosome spreads were prepared from fibroblast cell cultures obtained from muscle and connective tissues of female *E. velox* as described previously [48,49]. The W chromosome sample was microdissected from a Giemsa-stained metaphase plate, using an Olympus IX-51 microscope equipped with an Eppendorf Transferman NK2 micromanipulator, and amplified using the GenomePlex Whole Genome Amplification (WGA-1) kit (Sigma, Darmstadt, Germany). The lampbrush chromosome spreads were obtained from previtellogenic and early vitellogenic oocytes, using the standard avian lampbrush technique described by Saifitdinova et al. [50]. The total genomic DNA was extracted from ethanol-preserved blood, using the phenol–chloroform technique [51]. All manipulations with live animals and euthanasia were approved by the Saint Petersburg State University Ethics Committee (statement #131-03-2) and the Institute of Molecular and Cellular Biology Ethics Committee (statement #01/18 from 05.03.2018). 

The libraries of the W-specific probe and the total genomic DNA of a female *E. velox* were prepared using the TruSeq Nano DNA Low Throughput Library Prep Kit (Illumina, San Diego, USA) following the manufacturer’s protocol and sequenced on the Illumina MiSeq platform with the MiSeq Reagent Kit v3 (600 cycles). Before the sequencing, the libraries were examined for insert size and adapter content by electrophoresis in 2% agarose gel, and the molar concentrations were measured via qPCR. The sequence pre-processing steps included the trimming of Illumina adapters, WGA primers and low-quality bases, and discarding low-quality and too short (below 50 bp) reads with CLC Genomic Workbench (QIAGEN, Venlo, Netherlands) software. The sequencing reads were deposited in the NCBI SRA database under the accession number PRJNA716491.

Mapping the reads from the W-specific sample to the reference genome of *P. muralis* (GCF_004329235.1) was performed via Unipro UGENE v. 36 (https://www.ugene.net/, accessed on 1 September 2019) using the BWA-MEM algorithm [52] with default parameters and independently via the dopseq pipeline [53]. The regions of the reference genome with high coverage were searched via the UGENE graphical interface and using the dopseq output. The consensus sequences of the region of interest were extracted using both UGENE and the CLC Genomic Workbench. The total genomic DNA reads were aligned to the *P. muralis* genome as described above, and the previously identified sequences, which were present in the W-specific sample, were analyzed for coverage, and consensus sequences were extracted.

De novo assemblies of the W chromosome sample and the total DNA sample were generated via CLC Genomic Workbench using the ‘de novo assembly’ tool with default parameters. The W chromosome contigs were searched and aligned via the CLC Genomic Workbench using BLASTn search with the reference-based consensus sequence as query and the de novo assemblies as databases. Then, the identified W chromosome contigs were characterized via NCBI BLASTn through the web interface using the “*nr/nt*” database for all taxa and the “*refseq_genomes*” database with taxon specified as “Lacertidae” (i.e., the assemblies GCF_009819535.1 for *Lacerta agilis,* GCF_004329235.1 for *P. muralis*, and GCF_011800845.1 for *Zootoca vivipara*). The obtained W chromosome contigs were deposited in GenBank (MW846328-MW846339). To reconstruct the whole repeat unit, the identified contigs, which contain partial overlaps, were aligned to each other using CLC and MEGA X (https://www.megasoftware.net/, accessed on 1 October 2020) programs.

Since the data on lacertid repeated sequences is limited, RepeatModeler 2.0.1 [54] with the “-LTRStruct” option was used for the creation of a de novo library of repeated elements in the genomes of *L. agilis* (GCF_009819535.1), *P. muralis* (GCF_004329235.1)*,* and *Z. vivipara* (GCF_011800845.1). The resulting fasta files with classified repeats were clustered in a single file “lacertalib.fa” using cd-hit-est 4.8.1 [55] with default settings. Annotation of repeated DNA elements in the assembled contigs of *E. velox* using the de novo library obtained in the previous step was carried out using RepeatMasker 4.1.1 (http://www.repeatmasker.org/, accessed on 1 February 2021) with the parameters “-e rmblast -s -no_is -lib lacertalib.fa”.

To confirm that the identified sequences indeed belong to the W chromosome, we performed FISH with a PCR-amplified fragment of the reference-based consensus sequence (homologous to positions 46,390,515—46,391,408 of chromosome 14 in *P. muralis* assembly GCF_004329235.1). For this purpose, we designed the following primers: EVWF 5′-CCTGTAGGGTTCACCGTTCC-3′; EVWR 5′-GGGTTGAGTGACCTTCTCGG-3′. The primers were designed using the NCBI online tool Primer-BLAST (https://www.ncbi.nlm.nih.gov/tools/primer-blast/, accessed on 27 September 2019) based on the reference-based consensus sequence. We used the total genomic DNA of a female *E. velox* as a template. The PCR mix contained 10 μL of a PCR master mix (Biolabmix, Novosibirsk, Russia), 7 μL of MilliQ water, 1μL of 10 μM solutions of each primer, and 1 μL of the genomic DNA solution (50 ng/µL). The PCR conditions were as follows: initial denaturation at 95 °C for 5 min, 26 cycles of amplification (95 °C for 30 s, 55 °C for 30 s, 72 °C for 1 min), 72 °C for 5 min, and hold at 4 °C. The PCR results were checked by electrophoresis in 1% agarose gel. To prepare a fluorescently labeled amplicon, we carried out a secondary PCR round, using the amplicon as a template, with an addition of 1 μL of 1 mM TAMRA-dUTP (Biosan, Novosibirsk, Russia) to 20 µL of PCR solution. FISH with the amplified probe was carried out using standard techniques [56]. The preparations were visualized with an Axioplan 2 imaging microscope (Carl Zeiss, Oberkochen, Germany ) equipped with a CCD camera (CV M300, JAI), CHROMA filter sets, and ISIS4 image processing package (MetaSystems GmbH). The brightness and contrast of all images were enhanced using Corel PaintShop Photo Pro X6 (Corel Corp, Ottawa, ON, Canada).

## 3. Results

### 3.1. Reference-Based Assembly 

**Figure 1:**
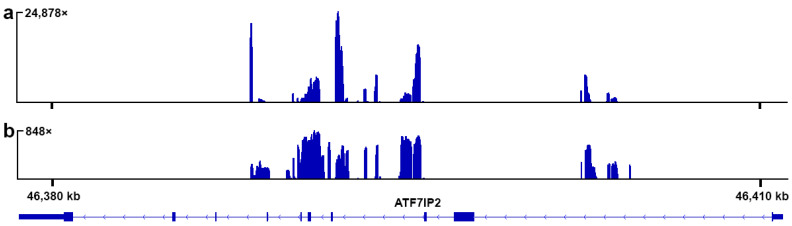
The distribution of coverage depth for the *E. velox* sequencing reads along the *ATF7IP2* gene of *P. muralis*. (**a**) The reads derived from the W-specific DNA sample; (**b**) the reads derived from the total genomic DNA sample. The Y axes indicate coverage. The X axis indicates the coordinates in the chromosome 14 scaffold of the *P. muralis* genome assembly (GCF_004329235.1). Exons are indicated as solid bars, introns are indicated by thin lines. The image is obtained via the Integrative Genomics Viewer (https://www.igv.org, accessed on 7 August 2019).

In total, 1,575,398 paired-end (PE) reads were obtained for the W-specific DNA sample and 2,797,722 PE reads for the total DNA sample. After the pre-processing, 859,598 PE reads and 1,828,412 PE reads were retained for the respective samples. The dopseq analysis of the W-specific sample reads revealed exceptionally high coverage (up to 24,878×, mean 3376×) of the fragments with a total length of 6170 bp inside a larger fragment of 17,213 bp length, situated inside the *ATF7IP2* gene on chromosome 14 (Supplementary Table S1, Figure 1a). The alignment of total DNA reads to *P. muralis* genome yielded high coverage in the same region, and although the maximum coverage was lower (up to 848×), the coverage graph was more even (Figure 1b). No significant homology between the W chromosome of *E. velox* and the Z chromosome was detected by the dopseq analysis.

### 3.2. De Novo Assembly

The de novo assembly statistics for the sequenced DNA samples were as follows: for the W-specific sample, N50 = 321, contig lengths 200–5386 bp, and 1438 contigs in total; for the total DNA sample, N50 = 360, contig lengths 200–9509 bp, and 250,177 contigs in total. From each assembly, we selected the contigs with the best BLAST alignment scores to the reference-based consensus sequence, which resulted in 2 sets of 6 contigs (12 contigs in total). The contigs from the W-specific sample assembly had a length distribution from 501 to 4126 bp and a total length of 11,035 bp. The contigs from the total DNA sample assembly had a length distribution from 485 to 3309 bp and a total length of 10,658 bp. 

**Figure 2:**
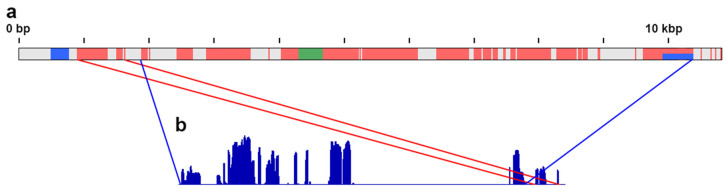
The reconstructed scheme of the *ATF7IP2*-derived repeat in the W chromosome of *E. velox*. (**a**) Sequence composition of the repeat unit. Pink indicates *ATF7IP2*-derived sequences, green indicates the POM/Squam1-SINE mobile element, blue indicates other SINE elements, and gray indicates sequences of unknown origin. (**b**) The reference-based scheme of the *ATF7IP2*-derived sequences (see Figure 1 for description), with their relative positions within the repeat unit indicated by the red and blue lines.

Mutual alignment of partially overlapping contigs from the two sets resulted in a final sequence 10,628 bp in length. Thus, the fragments derived from the *ATF7IP2* gene constitute a little more than half of the repeat unit, and other parts have different origins. The NCBI BLAST search did not retrieve significant hits for most of these non-*ATF7IP2* fragments, with few notable exceptions. The search in the lacertid genomic databases retrieved multiple hits for three small fragments of 200–400 bp. One of them is originally present in the *ATF7IP2* gene, whereas two are not (Figure 2a). The search in the “*nr/nt*” databases identified one of these non-*ATF7IP2* fragments as a SINE mobile element of the POM/Squam1 family, whereas two other repetitive fragments showed similarity to various unrelated lacertid microsatellite and mRNA sequences. The RepeatMasker search identified all three segments as SINE repeats. Interestingly, both assemblies had contigs that overlap both the beginning and the end of the reference-based sequence; however, there was a gap in this sequence that was not covered by any contigs (Figure 2b).

The *ATF7IP2*-derived fragments on the W chromosome of *E. velox* include both intron and exon sequences, although only 3 of the 10 original exons are present (Figure 1). The nucleotide analysis of the exon sequences showed one 3 bp insertion in one of the exons, in comparison with the reference genomes, but no premature stop codons.

### 3.3. FISH with the Probe to the Fragment of ATF7IP2

**Figure 3:**
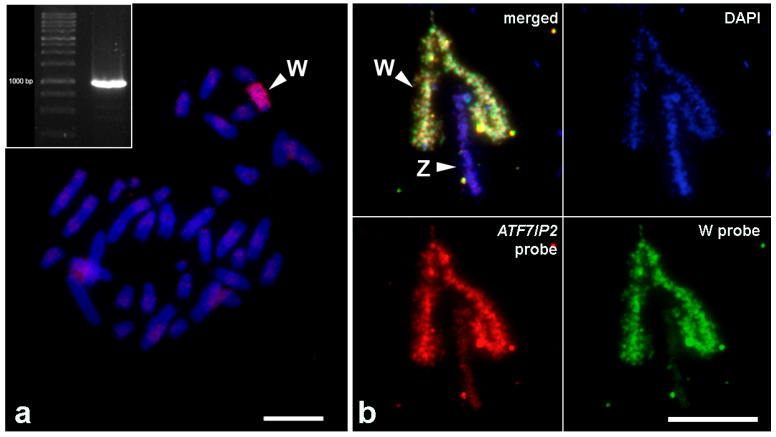
FISH with the PCR-amplified probe to the fragment of the *ATF7IP2* gene (red fluorescence) and the W-specific microdissected probe (green fluorescence). Chromosomes are counterstained with DAPI. (**a**) Metaphase plate of female *E. velox.* Scale bar: 20 μm. (**b**) Lampbrush ZW bivalent of *E. velox*. Scale bar: 15 μm. Insert: agarose gel electrophoresis with the PCR-amplified fragment of the *ATF7IP2* gene.

PCR amplification of the fragment of *ATF7IP2* resulted in a single band in the agarose gel, which corresponded to the predicted length. FISH with the probe derived from this amplicon resulted in an intense signal across almost the whole length of the W chromosome, except the centromeric region (Figure 3).

## 4. Discussion

Translocations of autosomal sequences onto sex chromosomes are widespread in vertebrates. In humans and other placental mammals, the sex chromosomes are constituted of the ancestral X/Y chromosome region and the added autosome-derived region [57]. In reptiles, such cases are known, for example, in geckos, anoles, and fence lizards [58,59,60,61]. In lacertids, the most well-known example is the European common lizard (*Z. vivipara*), which acquired a multiple sex chromosome system (Z_1_Z_2_Z_1_Z_2_/Z_1_Z_2_W) via a sex chromosome–autosome fusion [62]. However, all of these cases represent chromosomal rearrangements, involving whole autosomes or large autosomal fragments. The copying or repositioning of individual genes and their fragments, although predicted by the classical theory of sex chromosome evolution [7], is rarely documented. In the American crow, an intronless fragment of the gene *NARF* was copied to the W chromosome but was not amplified there [63]. In humans, the *DAZ* gene was copied to the Y chromosome and amplified in it, similarly to ATF7IP2 in *E. velox [64].* Three autosome-derived Y-linked genes were found in dogs [65]. Previously, we detected similar small autosome-derived segments in the sex chromosomes of anole lizards (*Ctenonotus sabanus* and *C. pogus*) by the dopseq analysis, but they were not studied in detail [66]. 

Here, for the first time, we minutely describe an autosome-derived sequence in a reptile sex chromosome, including its content and chromosomal localization. According to FISH, and based on high sequencing coverage, it is highly repetitive and dispersed throughout the whole W chromosome of *E. velox*, except its centromeric region. In lacertids, the centromeric heterochromatic blocks usually consist of centromere-specific satellites, which are common both to autosomes and sex chromosomes [40,67,68]. Thus, the centromeric region of the *E. velox* W chromosome is probably constituted of such a satellite, which is not related to the *ATF7IP2*-related repeat. In the main part of the W chromosome, different microsatellite sequences were previously localized [47]. Therefore, its sequence composition is quite complex, and the *ATF7IP2*-derived repeat is not the only repetitive sequence hosted in it. It was probably copied from the chromosome 14 and inserted to the W chromosome by a mobile element, and then amplified by subsequent mobile element activity or by intrachromosomal recombination. The absence of detectable homology between the W chromosome of *E. velox* and the Z chromosomes of reference lacertid genomes may be either because of a complete loss of any gametologous sequences or by the limited sensitivity of our method: since the W chromosome probe was prepared from a single microdissected chromosome, some parts of it may have been lost during microdissection and subsequent PCR amplification.

The reconstructed repeat unit is 10–11 kb long. The existence of contigs that span through the end and the beginning of the reference-based sequence suggests that it could be a tandem repeat, with “tail-to-head” organization. However, the gap inside the reference-based sequence, not covered by any contigs, suggests an alternative explanation: the *ATF7IP2*-derived sequences may be partially inverted or reshuffled in the repeat unit. Moreover, the different coverage in different parts of *ATF7IP2* suggests a divergence between the repeat units in their structure and composition. To reconstruct the exact structure and context of the *ATF7IP2*-derived repeat in the W chromosome of *E. velox*, its genomic DNA should be sequenced with a long-read technology, such as PacBio or Nanopore [69]. 

It is unknown whether the W chromosome copies of *ATF7IP2* fragments remain functional. The three preserved exons may potentially be translated, since they do not have stop codons, but it is unclear whether 3 of the 10 exons may still yield a functional product. Given the fact that the W chromosome of *E. velox* is transcribed, at least at the lampbrush stage, the identified *AFT7IP2*-derived repeat is the best candidate for the object of this transcriptional activity among the known W chromosome sequences. The proteins ATF7IP and ATF7IP2, also known as MCAF1 and MCAF2, are essential for trimethylation of lysine 9 in histone H3 and thus gene silencing and heterochromatin formation [70,71]. Thereby, if the W chromosome *ATF7IP2*-derived repeat is translated, its products may participate in chromatin regulation during oogenesis. Recently, non-coding RNAs derived from intronic sequences (sisRNAs, stable intronic sequence RNAa) were identified in the lampbrush-stage nuclei of a frog *Xenopus tropicalis,* and it was hypothesized that they could play important regulatory roles [72]. This suggests that the *ATF7IP2*-derived repeat could produce functional transcripts even if it does not encode a protein. Transcriptome and proteome analyses of lampbrush-stage oocytes of *E. velox* are required to clarify this question.

Insights into the possible functional significance of the *ATF7IP2*-derived repeat may also be gained from phylogenetic and evolutionary studies. The rapid racerunner is a widely distributed polymorphic species, which began to diversify around 6 MYA [73]. It would be interesting to study the W chromosome composition in different lineages of this species, as well as in other species of *Eremias*. Among the cytogenetically characterized *Eremias* species, *E. multiocellata* has a large heterochromatic W chromosome, similar to that of *E. velox* [74]. Another species, *E. arguta*, has a small W chromosome [41]. This suggests that the *ATF7IP2*-derived repeat may be shared by several species of *Eremias.* We demonstrated that it is easily found in low-coverage female genomic reads due to its high repetitiveness, and its fragments may be specifically amplified by PCR. This allows screening of the *Eremias* species for its presence even without cytogenetic analysis, via NGS or qPCR.

In conclusion, our results underline the complex structure and function of highly diverged Y and W chromosomes, which are often erroneously viewed as mere “degenerated” copies of X and Z chromosomes. In addition, our findings are a warning against making long-reaching general hypotheses on sex chromosome origins and evolution based on W and Y chromosome contents: we show that these chromosomes may accumulate sequences from other parts of the genome, unrelated to their origins.

## Data Availability

The sequencing reads were deposited in the NCBI SRA database under the accession number PRJNA716491. The obtained W chromosome contigs were deposited in GenBank (MW846328-MW846339).

## Supplementary Materials

The following are available online at https://www.mdpi.com/article/10.3390/genes12050779/s1, Table S1: The DOP-SEQ analysis of the sequences of the microdissected W chromosome probe of *Eremias velox*, aligned to the reference genome of *Podarcis muralis*.
